# A Model to Study the Phenotypic Changes of Insect Cell Transfection by Copepod Super Green Fluorescent Protein (cop-GFP) in Baculovirus Expression System

**DOI:** 10.7508/ibj.2016.03.008

**Published:** 2016-07

**Authors:** Narjes Shokrollahi, Delavar Shahbazzadeh, Kamran Pooshang-Bagheri, Mahdi Habibi-Anbouhi, Ali Jahanian-Najafabadi, Mahdi Behdani

**Affiliations:** 1 Biotechnology Research Center, Venom and Biotherapeutics Molecules Lab, Pasteur Institute of Iran, Tehran, Iran;; 2 National Cell Bank of Iran, Pasteur Institute of Iran, Tehran, Iran;; 3 Department of Pharmaceutical Biotechnology, School of Pharmacy, Isfahan University of Medical Sciences and Health Services, Isfahan, Iran

**Keywords:** Baculovirus, *Sf9*, Transfection

## Abstract

**Background:**

Baculovirus expression system is one of the most attractive and powerful eukaryotic expression systems for the production of recombinant proteins. The presence of a biomarker is required to monitor transfection efficiency or protein expression levels in insect cells.

**Methods:**

The aim of this study was to construct a baculovirus expression vector encoding a copepod super green fluorescent protein (copGFP). In this light, the resultant vector was constructed and used for transfection of *Spodoptera frugiperda* cells.

**Results:**

Expression of the copGFP protein in insect cells was confirmed by fluorescent microscopy and Western-blot analysis.

**Conclusion:**

The application of copGFP control bacmid can be considered as an appropriate control for insect cell transfection.

## INTRODUCTION

The application of recombinant proteins has increased dramatically in the recent years^[^^1^^]^. Nowadays, recombinant proteins are essential tools for studying biological processes^[^^2^^]^. There is a wide variety of protein expression systems, such as bacterial, mammalian, yeast, and insect cell. Baculovirus, one of the most commonly used expression systems, is an efficient tool for high-level expression of complex eukaryotic proteins, veterinary and human vaccines as well as biopesticides^[^^3^^-^^5^^]^. The *Autographa californica nucleo-polyhedrovirus* is an insect-specific virus that infects predominantly the larvae of the Lepidoptera order, but usually does not infect vertebrates^[^^2^^,^^6^^]^. Baculovirus expression vector is a recombinant virus genome engineered to clone the desired protein-coding sequence under the control of strong viral promoters, polyhedron, or P10^[^^7^^]^. In this study, we used Bac-to-Bac expression system that exerts Tn7 transposons for the production of recombinant baculoviruses. To monitor the transfection efficiency and expression levels of a given recombinant construct in a cell, plasmids containing reporter genes can be easily used. In the present study, we demonstrated that copepod super green fluorescent protein (copGFP), as a non-invasive, sensitive and convenient selectable marker, can be applied for direct measurement and detection of gene transfer efficiency in insect cells.

## MATERIALS AND METHODS

*Spodoptera frugiperda* (*Sf9*) cells were grown in Grace's insect cell medium (Invitrogen, USA) supplemented with 10% fetal bovine serum and 1% penicillin-streptomycin (Gibco, USA) at 27°C.

**Table 1:** The specific and universal primer sets used in this study

**Name**	Sequence
Nb-BacF	**5'-ACGGGATCCACAGGTGCAGCTGCAGGAGTCTGG-3'**
Nb-BacR	**5'-ACGCTCGAGTTATGAGGAGACGGTGACCTGG-3'**
Cop-BacF	**5'-ACGGGATCCGATGGAGAGCGACGAGAGC-3'**
Cop-BacR	**5'-ACGCTCGAGTTATTAGCGAGATCCGGTGG-3'**
polh-Forward	**5'-AAATGATAACCATCTCGC-3'**
PFSBC- Reverse	**5'-CCTCTACAAATGTGGTATGGC-3'**
pUC/M13-Forward	**5'-CCCAGTCACGACGTTGTAAAACG-3'**
pUC/M13-Reverse	**5'-AGCGGATAACAATTTCACAGG-3'**

**Fig. 1:**
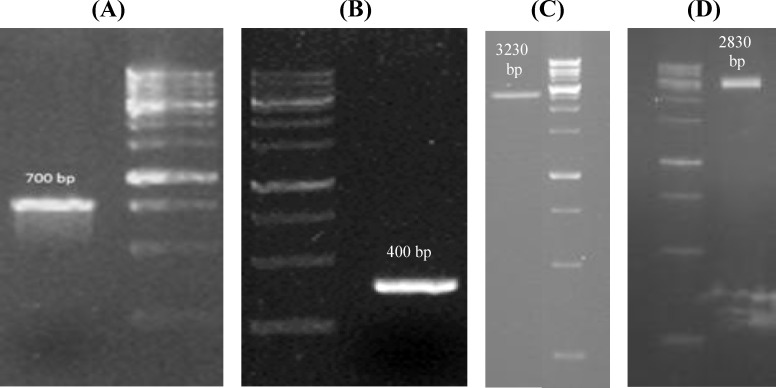
**PCR products analyzed on 1.5% agarose gel. As shown in the Figure, the size of copGFP (A) and 3VGR19 (B) PCR products are 700 and 400 bp, respectively. Colony PCR was performed using M13 universal primers on Bac-VGRNb (C) and Bac-copGFP (D) to confirm transposition into bacmid. The size of the DNA marker lanes from bottom to top is 250, 500, 750, 1000, 1500, 2000, 2500, 3000, 4000, 5000, 6000, 8000, and 10000 bp** **.**

VEGFR2-specific Nanobody (3VGR19) and copGFP genes were amplified from pHEN4-3VGR19 and pCDH-CMV-MCS-EF1-copGFP-T2A-Puro plasmids, respectively^[^^8^^]^. PCR was used to amplify the genes with specific primers containing *Bam*HI and *Xho*I restriction sites (Table 1). The PCR reaction was performed under standard conditions for 35 cycles (Fig. 1A and 1B). Then, the PCR products were cloned into a pFastBacHTA donor vector. The resulting constructs, pFast-VGRNb and pFast-copGFP, were transformed into the *E. coli* strain TOP10F' (Invitrogen, USA) and confirmed by colony PCR using universal primers (polh-Forward and PFSBC-Reverse) and DNA sequencing.

The purified plasmids, pFast-VGRNb and pFast-copGFP, were transformed into *E. coli *DH10Bac. To confirm transposition, colony PCR was performed using M13 universal primers (Table 1). As shown in Figure 1C and 1D, two bands, about 3230 bp (Bac-copGFP) and 2830 bp (Bac-VGRNb), were found after PCR amplification, and the recombinant bacmids were purified by the Carmo's method^[^^9^^]^. 

The recombinant bacmid was transfected into insect cells using the Bac-to-Bac baculovirus expression system (Invitrogen, USA) according to the manufacturer’s instructions. Briefly, 1 µg recombinant bacmid was dissolved in 100 µl Grace's insect cell culture. The resulting mixture was then mixed with 6 µl Cellfectin (Invitrogen, USA) reagent dissolved in 100 µl Grace's insect cell culture (Invitrogen, USA), after 20 min of incubation, 800 µl Grace's insect cell culture was added to the mixture, and subsequently transfected into 8×10^5 ^*Sf9* cells. The transfection mixture was then removed and replaced with a fresh growth medium containing 10% FBS and 1% penicillin-streptomycin in each well. Seventy-two hours post transfection, the signs of infection were observed under light and fluorescent microscopes. Transfected cells were analyzed after 48 h for visible hallmarks of *Autographa californica nucleopolyhedro-virus* infection. These hallmarks include cell growth arrest, cell size enlargement, and cell monolayer detachment.

**Fig. 2:**
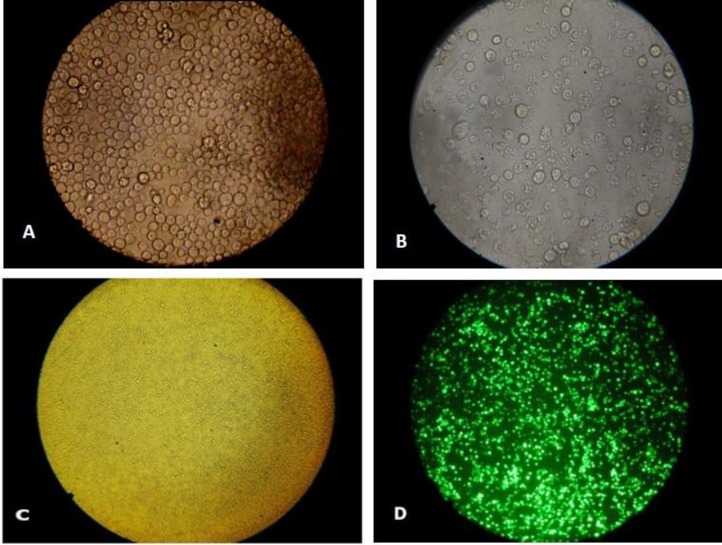
**R** **esult of transfection in** ***Sf9*** **cells. A) Control** **cells (×40); B) Cells transfected with Bac-VGRNb (×40); C) Control cells (×10); D) Cells transfected with Bac-copGFP (fluorescent microscope) (×10**

A large number of transfected cells carrying Bac-copGFP were found to have a bright green color, representing the successful transfection. Cell size enlargement and cell monolayer detachment were observed in the *Sf9* cells transfected with bacmid after three days. In addition, approximately 70% of the Bac-copGFP transfected cells exhibited a bright green color (Fig. 2). 

**Fig. 3:**
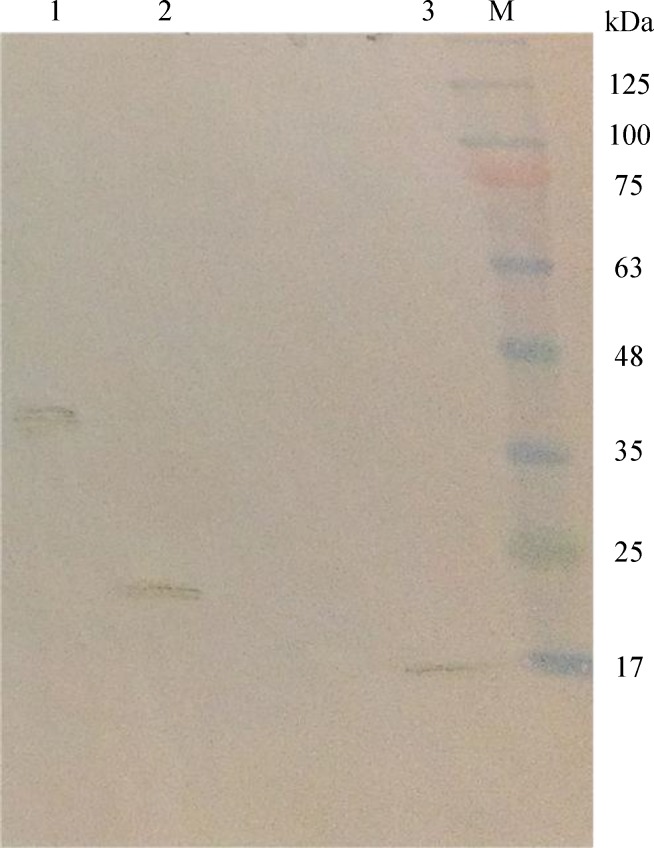
**Western-blot analysis for cell pellets obtained from** ***Sf9*** **cells transfected with recombinant baculovirus. Lane 1,** **copGFP (40 kDa); Lane 2, 3VGR19 (20 kSa); Lane 3, control protein (17kDa);** **M, marker**

After 72 hours post transfection, the supernatant containing virus particles was recovered by centrifugation at 664 ×g at 4°C for 3 min. The cell pellets were lysed by freeze-thaw cycles using liquid nitrogen and used for Western-blot analysis with the anti-His antibody. As shown in Figure 3, copGFP and 3VGR19 yielded 40- and 20-kDa bands, respectively.

## RESULTS AND DISCUSSION

In recent years, a variety of cell expression systems has gained increasing attention for their application in recombinant protein production. However, there seems to be a need for biomarkers to confirm the transfection process. In the case of transient transfection, there are a number of reporter genes, such as secreted alkaline phosphatase, β-galactosidase, firefly luciferase, and chloramphenicol acetyltransferase, which are used to detect and quantify transfection efficiency *in vitro*^[^^10^^]^. In the above-mentioned reporter genes, the gene transfer efficiency is indirectly assessed using the conditioned medium or cellular extracts of transfected cells. In addition, *in vivo* reporter assays, such as *in situ *β-gal staining, β-glucuronidase, and luciferase, are commonly used for transfection detection in either fixed cells or tissue sections. These methods allow the direct visualization of transfected cells after staining with enzymatic substrates or antibodies. In these assays, not only the presence of fixed cell cultures but also their reaction with substrates are required to detect transfected cells^[^^10^^]^.

GFP, as a reporter protein, is widely used to monitor gene expression and protein localization in a broad range of cells and organisms^[^^10^^,^^11^^]^. Several GFP-like proteins have been isolated from a number of copepod *aquatic crustacean* species^[^^11^^]^, the brightest of which has been made commercially available (Evrogen) under the name copGFP derived from copepod *Pontellina plumata*^[^^12^^,^^13^^]^. copGFP is characterized by superbright green fluorescence (at least 1.3 times brighter than EGFP), a fast maturation rate at a wide range of temperatures^[^^14^^]^, non-toxicity, high stability at a wide range of pH (pH 4-12), and no need for any additional cofactors or substrates^[^^11^^]^. During the years, GFP has been highly regarded as a biomarker in a wide variety of cells^[^^15^^, ^^16^^]^. 

In the current study, it was demonstrated that a reporter system containing the cop-GFP has a unique profile to monitor the transfection process in insect cells (*Sf9* cells). For this purpose, two recombinant baculovirus systems, containing copGFP as a biomarker and 3VGR19 as a control, were constructed to confirm the transfection procedure in insect cells. The present study clearly shows that the transfected cells can be easily detected using copGFP.

Following the transfection of insect cells with bacmids containing copGFP and 3VGR19, cytopathic effects were found in insect cells, and virions containing the gene of interest were released. In conclusion, the copGFP control bacmid (Bac-copGFP) can be used as a control for monitoring and optimizing transfection efficiency in insect cell transfection systems. 
